# Validation of a New Scintillating Fiber Dosimeter for Radiation Dose Quality Control in Computed Tomography

**DOI:** 10.3390/s23052614

**Published:** 2023-02-27

**Authors:** Nicolas Guillochon, Mamoutou Balde, Christian Popotte, Selena Pondard, Corentin Desport, Nicolas Kien, Fanny Carbillet, Ramiro Moreno, Mélodie Munier

**Affiliations:** 1 Fibermetrix, 7 Allée de l’Europe, 67960 Entzheim, France; 2 INSERM U1296 Radiations: Défense, Santé, Environnement, 69008 Lyon, France; 3 ALARA Expertise, 67960 Entzheim, France; 4 ALARA Group, 67960 Entzheim, France

**Keywords:** IVIscan, scintillation detector, optical fiber, dosimetry, CTDI, computed tomography, wide-beam, diagnostic

## Abstract

(1) Background: The IVIscan is a commercially available scintillating fiber detector designed for quality assurance and in vivo dosimetry in computed tomography (CT). In this work, we investigated the performance of the IVIscan scintillator and associated method in a wide range of beam width from three CT manufacturers and compared it to a CT chamber designed for Computed Tomography Dose Index (CTDI) measurements. (2) Methods: We measured weighted CTDI (CTDIw) with each detector in accordance with the requirements of regulatory tests and international recommendations for the minimum, maximum and the most used beam width in clinic and investigated the accuracy of the IVIscan system based on the assessment of the CTDIw deviation from the CT chamber. We also investigated the IVIscan accuracy for the whole range of the CT scans kV. (3) Results: We found excellent agreement between the IVIscan scintillator and the CT chamber for the whole range of beam widths and kV, especially for wide beams used on recent technology of CT scans. (4) Conclusions: These findings highlight that the IVIscan scintillator is a relevant detector for CT radiation dose assessments, and the method associated with calculating the CTDIw saves a significant amount of time and effort when performing tests, especially with regard to new CT technologies.

## 1. Introduction

The Computed Tomography Dose Index (CTDI) was introduced more than 40 years ago [1] and has facilitated government regulation and dosimetry standardization for computed tomography (CT) manufacturers [2]. It was developed to provide a standardized method to compare radiation output levels between different CT scanners. Today, it is also used for quality assurance purposes such as to monitor the deviation over time of CT scans in a relative way, and for optimization of patient radioprotection. It represents the average dose over a single slice in a standard cylindrical polymethyl methacrylate (PMMA) dose phantom of 15 cm in length, and 16 cm and 32 cm diameter for head and body examination, respectively. Theoretically, the CTDI should be measured from plus to minus infinity. Since in practice the pencil ion chamber to measure CTDI, called CT chamber, is typically 100 mm long, the International Electrotechnical Commission (IEC) has specifically defined the $CTDI_{100}$ measured in IEC 60601-2-44 Ed3.0 as

(1) $$CTDI_{100}=\int_{-50mm}^{+50mm}\frac{D\left(z\right)}{N\times T}dz,$$

where D(z) is a dose profile in air kerma along the longitudinal axis z. The number of detector channels and the width of each channel are N and T, respectively. N×T represents the total beam width.

The weighted computed tomography dose index, $CTDI_{w}$, which consists of a combination of central and peripheral measurements of $CTDI_{100}$ defined in Equation (1), in the PMMA dose phantom with applied weighted factors, corresponding to a linear decrease of the dose along the radial direction as

(2) $$CTDI_{w}=\frac{1}{3}\;CTDI_{100,center}+\frac{2}{3}\;\langle CTDI_{100,peripheral}\rangle ,$$

where $CTDI_{100,center}$ is the value measured at the center hole of the standard PMMA phantoms, and $\langle CTDI_{100,peripheral}\rangle$ is the average value measured at the four peripheral holes.

$CTDI_{100}$ and $CTDI_{w}$, were used in the past, but with the advent of modern scanners providing helical acquisitions, $CTDI_{vol}$ has been introduced and defined as the ratio between the $CTDI_{w}$ and the helical pitch. The pitch is generally defined as the ratio between the displacement of the table in one rotation of the gantry and the beam width. As all the acquisitions during mandatory controls are made in axial mode, the $CTDI_{vol}$ is rarely, if ever, used in the mandatory radiation dose quality control (RDQC) of CT scanners and is equal to the $CTDI_{w}$.

However, these indicators and the current method of obtaining them have major limitations and are not suitable for the new CT scan technologies [3,4] and tools such as wide-beam CT scanners. With the advent of multi-detector scanners, beam widths on the z-axis and the length of the scan have increased significantly. It has been shown on numerous occasions that for beam widths greater than 40 mm, the 100 mm CT chamber does not consider a large part of the primary and scattered radiation and the $CTDI_{100}$ underestimates the delivered dose by about 30–40% for a body phantom and 10–20% for a head phantom for the widest beams [5,6]. Today, CT scans can use beam widths up to 160 mm allowing to cover larger anatomical areas with clinical applications such as cardiac and head CT using the axial mode [7].

The IEC has described a two-tiered approach to the definition of CTDI. The first tier uses the conventional definition of $CTDI_{100}$ and is applied for beam widths < 40 mm. The second tier is used for beam widths > 40 mm.

In this new approach, it is assumed that the ratio between the $CTDI_{w}$ at two different beam widths is equal to the ratio of the $CTDI_{air}$, measured free-in-air, at these beam widths. Thus, it is proposed to calculate $CTDI_{w,N\times T>40}$, which is the weighted CT air kerma index for a beam width of N×T > 40 mm as follows:

(3) $$CTDI_{w,N\times T>40}=CTDI_{w,ref}\times \left(\frac{CTDI_{air,N\times T>40}}{CTDI_{air,ref}}\right),$$

$CTDI_{air,N\times T>40}$ and $CTDI_{air,ref}$ are the CT air kerma indexes measured free-in-air for a beam width > 40 mm and a beam width < 40 mm, respectively. $CTDI_{w,ref}$ is the weighted CT air kerma index for a reference beam width < 40 mm.

Several methods have been proposed to overcome this problem of dosimetry for wide beam CT scanners including small point detectors, multiple steps of a 100 mm CT chamber [8] or a 300 mm CT chamber [9,10,11]. Especially, the multiple steps method consists of multiple contiguous position measurements with step increments equal to the ion chamber length, i.e., 100 mm. However, this alternative method is rarely used because it is complex to set up and time consuming. On the other hand, the specific 300 mm CT chamber required for wide beams is quite fragile and expensive, and therefore it is rarely used. The small ionization chambers are dedicated to radiotherapy and so need a specific calibration for CT dosimetry. Solid-state detectors might suffer from energy and angular dependence so their use in CT application for CTDI measurements is not really appropriate.

It is clear that even though the regulatory texts and international recommendations require that radiation dose evaluation be performed under conditions as close as possible to clinical use [12]; in practice, the wide beams are still not always verified today. Indeed, RDQC should have meaningful results using a quick measurement, and be accessible and reproducible.

The Fibermetrix® company has developed a measurement method and associated device named IVIscan®, based on a plastic scintillating fiber sensor of two meters long [13,14]. Devic et al. have mainly characterized the system and demonstrated its accuracy in terms of air kerma measurements in the CT scan energy range [15]. Scintillating optic fiber has been under development for several years for medical dosimetry applications [16,17,18] and is based on various materials such as doped silica fiber or plastic scintillating fiber. The Exradin W1 and W2 (Standard Imaging Inc., Middleton, WI, USA) [19,20,21] have especially been developed for radiotherapy applications and have been marketed for a few years. The IVIscan real-time dosimeter is an in vivo dosimetry solution dedicated to medical imaging. It is the first commercially available dosimetry solution that is permanently placed on the CT table, operates in full autonomy, and could have applications in RDQC [22,23]. Moreover, the two meter sensor length allows the integration of the dose even on wide-beams acquisition and all along the explored length in clinical examinations.

This study evaluates dosimetric performance of this scintillating fiber detector as part of the CT scan RDQC with a particular focus on detector response in wide-beam scanners. So, we compared the IVIscan dosimeter and the method associated with a reference CT chamber and validated it as a new fiber dosimetry system for RDQC. We proposed to calculate the $CTDI_{w}$ and the $CTDI_{w,N\times T>40}$ defined in Equations (2) and (3) by the two measurement methods, in regulatory conditions on multiple CT scans for thinnest, largest and usual beam widths used in clinical practices and beyond for the different X-ray tube voltages (kV). The kV variation allows us to study the energy dependency of the IVIscan detector. Then we evaluated the relative deviation between the CTDIw obtained through the reference method using the reference CT chamber, $CTDI_{w}^{chamber}$, and the one obtained through the IVIscan method, $CTDI_{w}^{IVIscan}$.

## 2. Materials and Methods

### 2.1. Computed Tomography Equipment

In order to validate IVIscan dosimeter in a universal way, measurements of all $CTDI_{w}$ were carried out with four different CT scans from the three main CT manufacturers. A Siemens SOMATOM® definition AS+ called System Number 1 (SN1), a Canon Medical Aquilion ONE Genesis (SN2), a GE Healthcare Revolution CT (SN3) and a Canon Medical Aquilion ONE/PRISM Edition (SN4). SN2, SN3 and SN4 have beam widths over 40 mm while SN1 has traditional beam widths of less or equal to 40 mm. The highest voltage used by the CTs was 140 kV, with the exception of the SN2 and SN4 which use 135 kV.

### 2.2. Irradiation Parameters

CTDIw calculation from RDQC tests requires specific CT acquisition parameters as tube voltage and beam width. Thus, the aim was to first validate the IVIscan dosimeter under these specific requisites. So, the reference and IVIscan measurements were performed at 120 kV, at the thinnest and largest beam width used in clinic, for a head and body protocol with the corresponding phantom, as required by the French regulation related for the quality control of CT scanners [24]. Medical physics experts are unanimous that RDQC should be more closely aligned with clinical practice, especially with respect to the protocols used. Thus, we carried out measurements at the most common beam width used in clinical routine too. For each case, we settled an X-ray tube current of 200 mA and a tube rotation of 1 s, in order to adapt the dose rate to accurate measurements with the CT chamber, which is the reference in this study.

In addition, we evaluated the IVIscan dosimeter repeatability in terms of CTDIw calculation on a wide range of dose rate and beam qualities available on CT scans. For this purpose, IVIscan measurements were carried out on the entire range of SN1 tube voltage, from 70 to 140 kV, and two beam widths of 5 mm and 38.5 mm. Assuming that the protocol type, head or body, has less impact on the dosimeter repeatability than the tube voltage or dose rate, we chose only head protocols for this test.

We also wanted to investigate the energy dependence of IVIscan dosimeter. In this way, further measurements were carried out beyond the RDQC requisites with the CT chamber and IVIscan dosimeter, for the whole range of SN2 tube voltage, i.e., 80, 100, 120 and 135 kV, and beam widths of 2 mm and 40 mm. Given the high number of measurements to be carried out with the CT chamber, we also considered that it was sufficient to carry out these tests with a single protocol type. Thus, we chose body protocol.

### 2.3. Reference Dose Indexes

#### 2.3.1. Reference Materials

To measure the parameter of CT radiation dose, the study was conducted using a 100 mm Unfors Raysafe^TM^ X2 CT chamber (Fluke Biomedical, Everett, WA, USA) and standard-sized CTDI PMMA head and body phantoms. The CT chamber was calibrated according to IEC 61223-2-6. Measurements were made with an uncertainty of less than 5% at the 95% confidence level (manufacturer data).

The two standardized PMMA cylindrical phantoms of 16 cm diameters and 32 cm diameters were used for head CT protocols and body CT protocols, respectively. Both phantoms are 15 cm in length and have five 12.4 mm diameter holes. One of them is located at the center and the other four, 10 mm beneath the surface at 90° intervals.

#### 2.3.2. Reference Measurements

- CT scan Compliance

Firstly, an evaluation of the conformity of the four scanners was carried out by a primary RDQC with a CT chamber in standard quality control conditions. According to international regulation, the $CTDI_{vol}$ value displayed by the scanner must not deviate by more than ±20% from the measured $CTDI_{w}^{chamber}$ [25]. Then the relative deviation between $CTDI_{vol}$ displayed by the CT scan and $CTDI_{w}^{chamber}$ was defined in percent as

(4) $${\left[\Delta CTDI\right]}_{ref}^{CT}=\left(\frac{CTDI_{vol}-CTDI_{w}^{chamber}}{CTDI_{w}^{chamber}}\right)\times 100,$$

$CTDI_{vol}$ is calculated by dividing $CTDI_{w}$ with the pitch. Since all measurements are made with axial acquisitions with pitch = 1, $CTDI_{vol}$ is equal to $CTDI_{w}$.

- Beam Width < 40 mm

**Figure 1:**
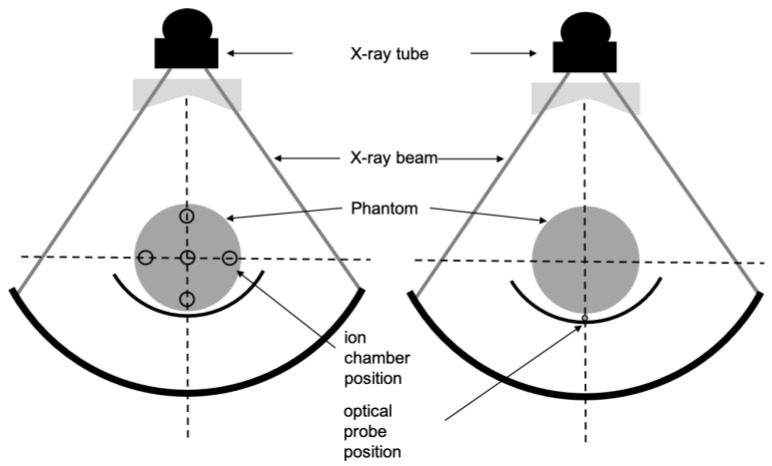
Diagram demonstrating practical measurement of weighted CT air kerma index ($CTDI_{w}$) for beam width < 40 mm with a 100 mm CT chamber (**left**) and IVIscan detector (**right**). The phantom is placed on the CT table in both cases.

$CTDI_{w}^{chamber}$ was calculated according to Equation (2) defined above from the five measurements in the phantom holes. Figure 1 shows the practical measurement of CTDIw for beam width < 40 mm. The CT chamber setup is on the left side on the figure. The evaluation was performed over the thinnest beam width (2 mm for SN2 and SN4 and 5 mm for SN1 and SN3) and the most used beam width for clinical protocols (38.5 mm for SN1 and 40 mm for SN2, SN3, and SN4). A width of 12 mm was tested for SN4 as well.

- Beam Width > 40 mm

**Figure 2:**
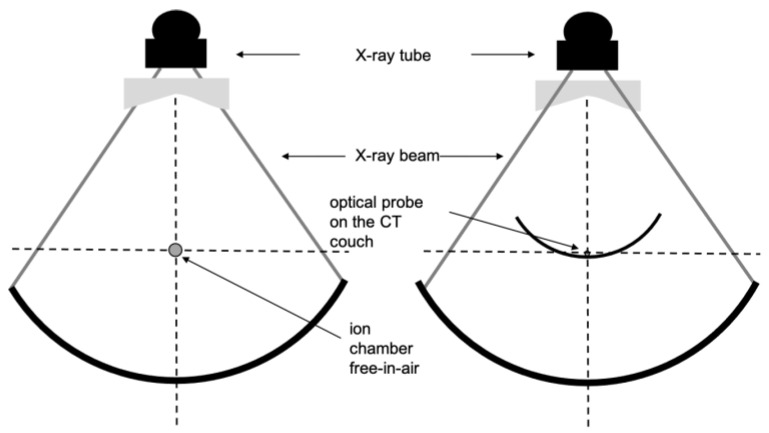
Diagram demonstrating practical measurement of the CT air kerma indexes measured ($CTDI_{air,N\times T>40}$) for beam width N×T larger than 40 mm with a 100 mm CT chamber free-in-air (**left**) and with IVIscan detector on the CT table (**right**). No phantom is used here.

**Figure 3:**
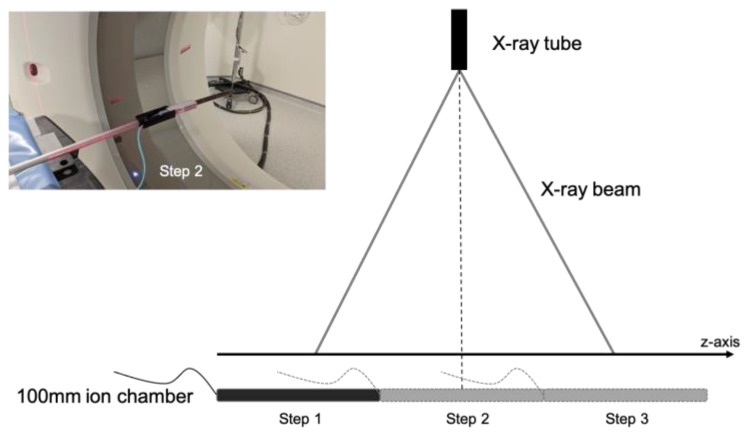
Diagram representation of the recommended three-step in-air measurement method of $CTDI_{air,N\times T>40}$ for beam width N×T larger than 40 mm with a 100 mm CT chamber.

To perform CTDI calculation with beam widths over 40 mm, we used the International Atomic Energy Agency (IAEA) method described in the paper introduction and using the same CT chamber as for beam width < 40 mm. The evaluation was performed over the largest beam widths from 80 to 160 mm according to the CT scan technical possibilities. The CT chamber was then attached to a retort stand on the CT table and aligned at the isocenter of the beam as shown in Figure 2 (left). The table was then stepped through in the z-direction in increments of 100 mm and a beam rotation was performed at each increment step. The CT chamber was placed away from the edge of the table to avoid any scattered radiation from the table. Dose measurements were carried out in the three sequential steps as shown in Figure 3, and summed to give $CTDI_{air,N\times T>40}$. Then, $CTDI_{w,N\times T>40}^{chamber}$ was calculated according to Equation (3) defined above with a reference beam width of 40 mm.

### 2.4. IVIscan Dose Indexes

#### 2.4.1. IVIscan Scintillating Fiber Detector

**Figure 4:**
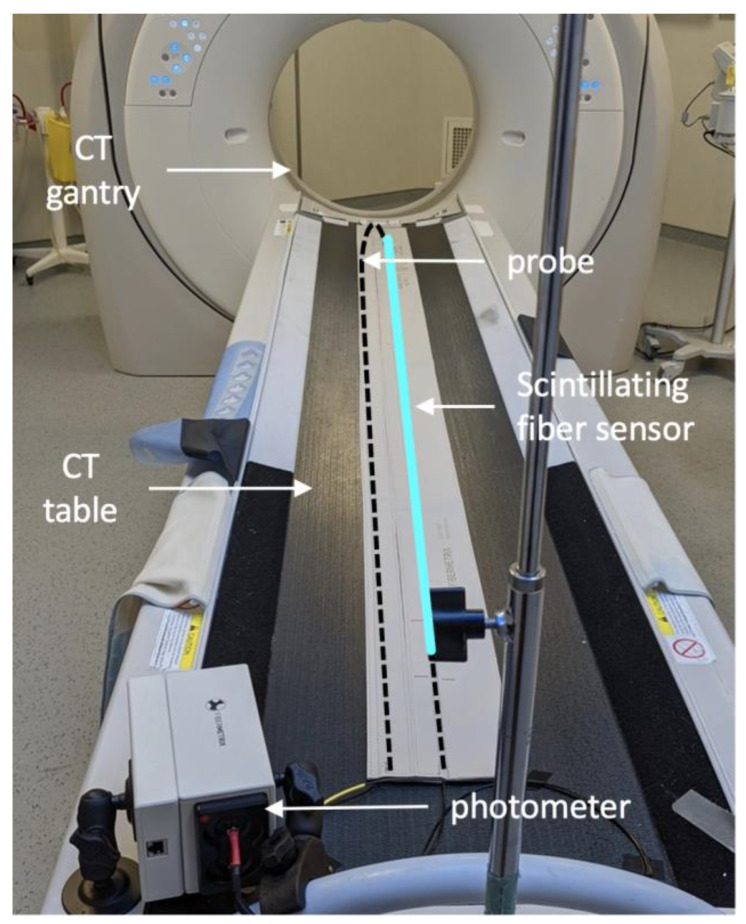
IVIscan system installed on a CT scan. The probe is placed on the CT table forming a U-shape at the head side of the table (black dotted line) and is connected to the photometer fixed at the feet side of the table. The scintillating fiber sensor (blue line) is placed in line to measure air kerma all along the exposure area on the CT table.

The IVIscan® (Fibermetrix®, Entzheim, France) is a commercially available dosimeter dedicated to automatic dose measurements and dose index calculations on CT scans [26]; it is composed of a two-ended scintillating probe and an electronic part, named the photometer, detecting the light emitted by the probe. The probe consists of a scintillating plastic fiber sensor of 2 m long connected to a clear plastic optical fiber, acting as a light guide, at each sensor end. The total probe length is thus about 6 m. The material composition of the probe has been described in previous work [15]. It is permanently installed under the CT couch forming a U-shape at the head side of the table (Figure 4) and thus, it is not visible. The 2 m sensor is longer than the maximum exposed length on the CT table, and therefore it is possible to carry out dose measurements everywhere on the CT table. The probe is connected to the two-channel photometer, which measures the scintillation light emitted by the probe due to ionizing radiation interactions with the scintillating fiber and converts it into radiation dose. IVIscan dosimeter was calibrated in terms of air Kerma in RQT9 beam quality, which is the reference beam quality in scanography. It was characterized according to IEC 61674 at the Laboratoire National Henri Becquerel (LNHB, CEA LIST, Saclay, France), an independent primary calibration laboratory. A software solution is permanently connected to the dosimeter and the CT scan both to recover the measured data and all the implementation parameters of the CT scan for each irradiation protocol. History of dose measurements and RDQC is available and a quality control report can be easily generated.

Due to the high variability in CT beam quality depending on the examination protocols (i.e., tube voltage and beam filtration), the calibration factor $N_{k,RQT9}$ is automatically corrected by the software during the installation of the dosimetry system on each CT scan to offset the energy dependence and give the most accurate dose D(z). This correction method was also detailed in the previous work cited above. Air kerma, and hence dose measurements, were made with an uncertainty of less than 5% at the 95% confidence level (manufacturer data).

#### 2.4.2. IVIscan Measurement Method

For beams less or equal to 40 mm, the method for calculating $CTDI_{w}$ with IVIscan dosimeter was carried out with only one measurement instead of the five with a CT chamber, i.e., one for each phantom hole, required for the usual $CTDI_{w}$ calculation (Figure 1). A single dose measurement, $CTDI_{2000table}$, was performed on the table, under the phantom, for a 360° X-ray tube rotation. A specific conversion factor $N_{C}$ was then automatically applied by the processing module and the beam width was automatically taken into account to obtain the $CTDI_{w}^{IVIscan}$ such as

(5) $$CTDI_{w}^{IVIscan}=CTDI_{2000table}\times N_{C},$$

It is to be noted that $CTDI_{2000table}$ is the dose D(z) measured over the 2000 mm sensor length. The conversion factor $N_{C}$ is selected from a conversion factor table stored in the memory of the device according to the CT scan acquisition type and basic radiation-matter interaction rules. The table is generated for each CT scan by a set of initial measurements during the commissioning of the dosimeter so as to take into account the specificity of each CT scan. The $N_{C}$ factors are acquired for head and body protocols at 120 kV and one beam width only, usually 10 mm. The method of obtaining $N_{C}$ factors is not detailed in this work. This is largely described in the Fibermetrix patents [27,28].

For beams over 40 mm, the method involves the measurement of $CTDI_{2000table,\;N\times T>40}$ which is a dose measurement on the table too but without any phantom. Then, according to Equation (5):

(6) $$CTDI_{w,N\times T>40}^{IVIscan}=CTDI_{2000table,N\times T>40}\times N_{C^{*}},$$

where $N_{C^{*}}$ is another specific conversion factor stored in the memory of the device and depending on the anatomical type of the irradiation protocol (head or body). Compared to Equation (3), this conversion factor takes into account the absence of any phantom and the measurement on the CT table instead of free-in-air as for the IAEA methodology for wide-beam RDQC.

Then, the $CTDI_{w,N\times T>40}$ was calculated from a single measurement on the table with the IVIscan method, instead of the multiple measurements in air and in phantom holes required for CTDI calculation with a 100 mm CT chamber. To compare the results between IVIscan and the reference CT chamber methods as accurately as possible, the scintillating fiber sensor measurements were carried out together with the reference CT chamber so that there is no additional uncertainty associated with the X-ray emission or another environmental parameter.

### 2.5. Statistical Analysis

#### 2.5.1. Repeatability

A first step of $CTDI_{2000table}$ and $CTDI_{2000table,N\times T>40}$ measurements was carried out to evaluate the repeatability of the IVIscan measurement method. Mean $CTDI_{w}^{IVIscan}$ and $CTDI_{w,N\times T>40}^{IVIscan}$, and relative standard deviation (RSD) were calculated on the basis of three replicates on SN1.

#### 2.5.2. Energy Dependence

The relative deviation of the CTDIw between the scintillating fiber sensor and the reference CT chamber was calculated in RDQC conditions according to the following equation:

(7) $${\left[\Delta CTDI\right]}_{ref}^{IVIscan}=\left(\frac{CTDI_{w}^{IVIscan}-CTDI_{w}^{chamber}}{CTDI_{w}^{chamber}}\right)\times 100,$$

Lastly, mean ${\left[\Delta CTDI\right]}_{ref}^{IVIscan}$ and RSD associated were calculated on the basis of three replicates on SN2 for the four available tube voltages.

## 3. Results

### 3.1. CT Scan Compliance

**Figure 5:**
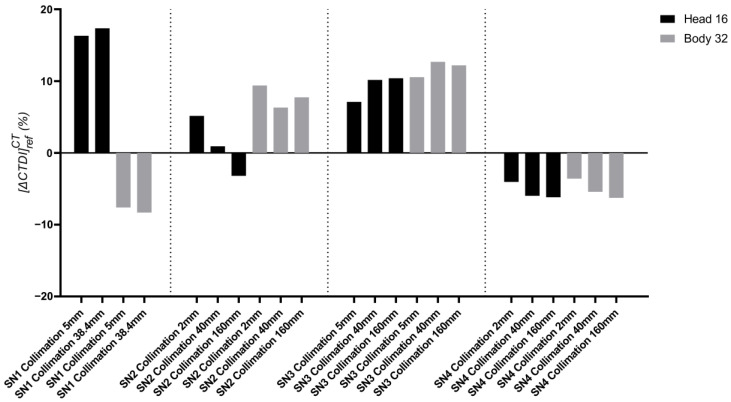
Relative deviation between $CTDI_{w}$ displayed by the CT scan and CT chamber, ${\left[\Delta CTDI\right]}_{ref}^{CT}$, for standard 120 kV radiation dose quality control (RDQC) performed on a Siemens SOMATOM® definition AS+ (SN1), a Canon Medical Aquilion ONE Genesis (SN2), a GE Healthcare Revolution CT (SN3) and a Canon Medical Aquilion ONE/PRISM Edition (SN4). The measurements were performed at the minimum, maximum and most used beam widths for head and body protocols.

Figure 5 shows the deviation ${\left[\Delta CTDI\right]}_{ref}^{CT}$ between $CTDI_{w}$ displayed by the CT scan, and $CTDI_{w}^{chamber}$ and $CTDI_{w,N\times T>40}^{chamber}$ measurements using the 100 mm CT chamber for a standard RDQC performed on the four CT scanners SN1, SN2, SN3 and SN4. Both head and body protocols were controlled each time for the thinnest, largest and most beam width used in clinic.

A great disparity from −6.26% to +17.4% is observed between the different CT scanners, the different beam widths and the different phantoms used. The highest deviations are observed for SN1 for the head phantom at 5 mm and 40 mm beam width with 16.3% (±0.64) and 17.4% (±0.46), respectively. However, the deviation does not exceed ±20%, so the four CT scanners SN1, SN2, SN3 and SN4, meet the criteria for acceptable RDQC and can be used in this study.

For the remainder of this study, the $CTDI_{w}$ displayed by the CT scan will not be considered. Only the results obtained with the 100 mm CT chamber and IVIscan dosimeter will be compared.

### 3.2. IVIscan Validation

#### 3.2.1. Repeatability

First, Table 1 shows the mean $CTDI_{w}^{IVIscan}$ obtained with the IVIscan method and the relative standard deviations associated for head protocols at 70, 100, 120 and 140 kV SN1 tube voltages, and 5 mm and 38.5 mm beam widths. The relative standard deviation is between 0.02% for 140 kV and 38.5 mm beam width and 0.21% for 70 kV and 5 mm beam width. We characterized the IVIscan repeatability in terms of air kerma in reference beam qualities in a previous study [15], and demonstrated that the specific uncertainty was about 0.02% for dose rate close to the minimum dose rates used in CT (<1 mGy). So, we can reasonably assume that the relative standard deviations (RSDs) observed here are mainly due to the stability of the CT X-ray emission, and especially for low dose rates. However, these results indicate very good repeatability with the scintillating fiber sensor methodology.

#### 3.2.2. Deviation between $CTDI_{w}^{IVIscan}$ and $CTDI_{w}^{chamber}$

**Figure 6:**
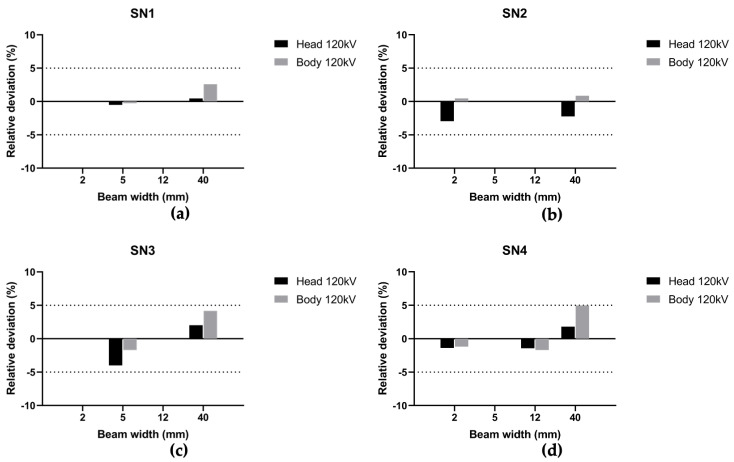
Relative deviation between $CTDI_{w}^{IVIscan}$ and $CTDI_{w}^{chamber}$, ${\left[\Delta CTDI\right]}_{ref}^{IVIscan}$, at 120 kV for head and body phantoms over the thinnest and the most used beam width in clinic for (**a**) SN1, (**b**) SN2, (**c**) SN3 and (**d**) SN4.

**Figure 7:**
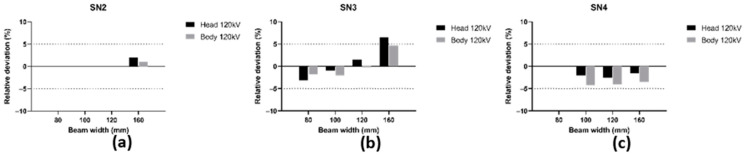
Relative deviation ${\left[\Delta CTDI\right]}_{ref}^{IVIscan}$ at 120 kV for head and body phantoms over the largest beam width for (**a**) SN2 with N×T = 160 mm, (**b**) SN3 with N×T = 80, 100, 120 and 160 mm, (**c**) SN4 with N×T = 100, 120 and 160 mm.

**Figure 8:**
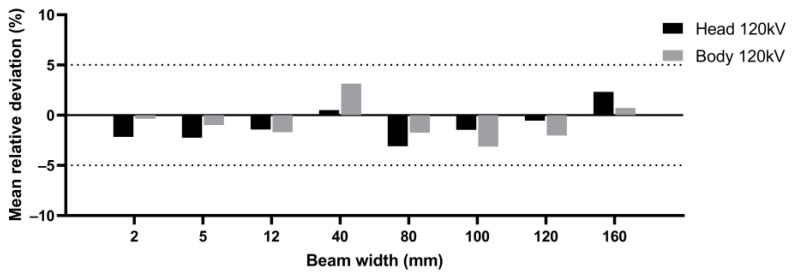
Mean ${\left[\Delta CTDI\right]}_{ref}^{IVIscan}$ over all centers at 120 kV for each protocol type (head and body and beam width from 2 mm to 160 mm).

Then, we compared the two different methodologies for RDQC on the four different CT scans to validate a new scintillating fiber dosimeter and to assess the corresponding accuracy. Hereafter in this paper, the beam width of 38.5 mm for SN1 is treated as a 40 mm beam width to compare results with other CT scans. The results for each CT scan are displayed in Figure 6 for beam width < 40 mm and Figure 7 for beam width > 40 mm. Figure 8 shows the mean ${\left[\Delta CTDI\right]}_{ref}^{IVIscan}$ over all CT scans by protocol type, i.e., beam width and anatomic region of head and body.

Regarding beam width < 40 mm, results presented show a relative deviation between −3.9% and +4.9%. The maximum value relates to SN4, 40 mm beam width and body protocol (Figure 6d). Considering all measurements, the relative deviation does not exceed ± 5% regardless of the CT scan and protocol used and the mean ${\left[\Delta CTDI\right]}_{ref}^{IVIscan}$ ranges from −2.25% to +3.14% according to the CT protocol type (Figure 8). In view of the respective uncertainty of the two dosimeters, we can conclude that the two methodologies of calculating the $CTDI_{w}$ are equivalent for RDQC with beam < 40 mm, and the $N_{C}$ factor is independent of the beam width.

Similar results are obtained for beam width > 40 mm except for SN3, at 160 mm, and a head protocol where a relative deviation of +6.5% is obtained. In all other cases, the relative deviation is between −4.2% and +4.6% and thus does not exceed ±5%. The mean ${\left[\Delta CTDI\right]}_{ref}^{IVIscan}$ shown in Figure 8 is between −3.13% and +2.33% according to the CT protocol type. Therefore, we can reasonably conclude here as well that both methodologies of calculating the $CTDI_{w,N\times T>40}$ for a beam over 40 mm are equivalent.

#### 3.2.3. Energy Dependence

**Figure 9:**
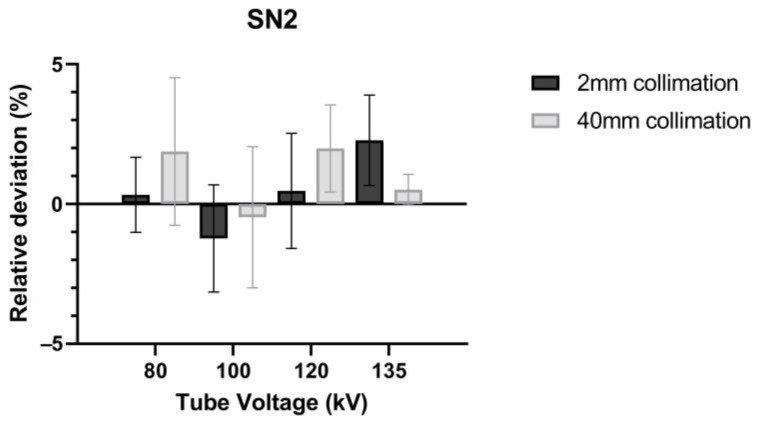
Relative deviation ${\left[\Delta CTDI\right]}_{ref}^{IVIscan}$ calculated for SN2 Head CT protocols over the thinnest beam (2 mm beam width) and the most used in clinic (40 mm beam width) for the entire CT energy range. The standard deviations are represented as error bars.

Even though the regulations require RDQC to be performed at a fixed energy of 120 kV, we wanted to study the relative deviation ${\left[\Delta CTDI\right]}_{ref}^{IVIscan}$ on the entire energy range of a CT scan. Figure 9 shows the results obtained for SN2 at 80, 100, 120 and 135 kV for body CT acquisition and two different beam widths.

Relative deviation ${\left[\Delta CTDI\right]}_{ref}^{IVIscan}$ ranges from −1.23% (±1.92) to +2.28% (±1.62) and does not exceed ±5% regardless of the beam width used. Therefore, we can assume that the IVIscan method does not depend on the beam quality. This makes it possible to carry out RDQC for different kV parameters with the same accuracy, especially for the specific control of pediatric protocols which are often 100 kV or high body mass index (BMI) protocols which are often 140 kV.

## 4. Discussion and Conclusions

As CT is the most radiative imaging modality, it is important to be able to accurately estimate the output radiation and ideally in the conditions of clinical use. Today, the $CTDI_{w}$, is the reference indicator used for RDQC. However, this indicator suffers from several limitations. First, 100 mm CT chamber and PMMA phantom lengths do not allow for accurate measurement of the larger beam width available on the new wide beam CT scans. The current tools and methods are therefore obsolete in addition to being time consuming which may interfere with clinical activity. According to current French and international regulations, RDQC is only carried out once a year and as an internal control when changing an X-ray tube or any hardware of software intervention that may influence the X-ray emission. This can lead to the late detection of a dosimetric mismatch. Therefore, a simplified and automated method allowing quick and relevant measurements, without impacting the clinical activity, is needed and could encourage more regular controls.

The results presented in this work show a good correlation between IVIscan and the usual CT chamber method, no matter which scanner, phantom, energy or beam width is used. We observed only one relative deviation above +5%, and it represents a minority of the total of 34 measurements, comprising 16 measurements over 40 mm beam width. Moreover, considering the ±5% uncertainty related to each dosimetry system and the accuracy of the phantom positioning for each comparative test, it can be assumed that these results highlight an equivalence of the IVIscan method and associated scintillating fiber sensor and usual CT chamber for CTDIw measurements. Thus, these results are very satisfactory and validate the use of the IVIscan® dosimeter within the framework of standard regulatory RDQC in computed tomography. Complementary results with kV variation show the non-dependency of IVIscan methodology with the beam energy that is convenient for performing quality controls closer to the clinic.

The competent authorities are currently considering changing the quality control procedures to address the various issues outlined above and thus come closer to clinical practices. With its sensor shape and radiolucency, allowing measurements during clinical examinations, and its detection length, the IVIscan device would overcome these difficulties both for wide beams and helical acquisitions which make it possible to imagine RDQC that are close to the current clinical practices in CT.

Furthermore, the IVIscan detector and associated methods described in this article could find interesting applications in other fields (e.g., radiotherapy) with the use of Cone Beam CT (CBCT) imaging equipment, which has the particularity to also have a wide beam. This imaging equipment is increasingly growing in the field of image-guided radiotherapy. It has been henceforth recommended by multiple international institutions to report the imaging dose given through radiotherapy treatment courses and implement a quality control system able to control the X-ray tube performances over time. Since there is no standardized approach to CBCT quality control and dosimetry across all CBCT systems, and the measurement of CTDI on CBCT is very time-consuming, it could be interesting to study the relevance of the IVIscan dosimeter for RDQC on this kind of device. This will be the subject of future work.

## Figures and Tables

**Table 1:** Mean $CTDI_{w}$ measured with IVIscan, $CTDI_{w}^{IVIscan}$, and relative standard deviation (RSD) associated for three replicates on SN1 for head protocols at 70, 100, 120 and 140 kV and for beam widths of 5 mm and 38.5 mm.

Beam width (mm)	5				38.5			
Tube voltage (kV)	70	100	120	140	70	100	120	140
Mean $CTDI_{w}^{IVIscan}$ (mGy)	3.78	12.77	21.52	32.19	4.31	14.43	24.11	35.70
RSD (%)	0.21%	0.07%	0.10%	0.16%	0.05%	0.11%	0.10%	0.02%
